# Effects of Different Dietary β-Glucan Levels on Antioxidant Capacity and Immunity, Gut Microbiota and Transcriptome Responses of White Shrimp (*Litopenaeus vannamei*) under Low Salinity

**DOI:** 10.3390/antiox11112282

**Published:** 2022-11-18

**Authors:** Yanbing Qiao, Li Zhou, Yayu Qu, Kunyu Lu, Fenglu Han, Erchao Li

**Affiliations:** Key Laboratory of Tropical Hydrobiology and Biotechnology of Hainan Province, Hainan Aquaculture Breeding Engineering Research Center, College of Marine Sciences, Hainan University, Haikou 570228, China

**Keywords:** *Litopenaeus vannamei*, aquaculture, low salinity, β-glucan, transcriptome

## Abstract

β-Glucan could significantly improve the antioxidant capacity of aquatic animals. The effects of different dietary levels (0 (control), 0.05, 0.1, 0.2 or 0.4%) of β-glucan on the growth, survival, antioxidant capacity, immunity, intestinal microbiota and transcriptional responses of *Litopenaeus vannamei* under low salinity (≤3) were investigated. The dietary growth trial lasted 35 days (initial shrimp 0.26 ± 0.01 g). The results indicated that the growth performance of the 0.1% and 0.2% groups was significantly better than that of the control group. A second-order polynomial regression analysis of growth performance against dietary β-glucan indicated that the optimal dietary β-glucan level was 0.2% of dry matter. The digestive enzyme activity of the hepatopancreas was enhanced with increasing β-glucan levels. The antioxidant and nonspecific immunity capacities of the hepatopancreas were also enhanced in the 0.1% group. The α-diversity index analysis of the intestinal microbiota showed that the intestinal microbial richness of *L. vannamei* increased in the 0.1% group. The relative abundance of Proteobacteria decreased in the 0.1% group compared with the control group. The transcriptome results indicate that the prebiotic mechanisms of β-glucan include upregulating the expression of nonspecific immune genes and osmoregulation genes and activating KEGG pathways associated with carbohydrate metabolism under low-salinity stress. These results suggested that dietary supplementation with β-glucan markedly increased growth performance and alleviated the negative effects of low-salinity stress by contributing to the activity of biochemical enzymes and enriching carbohydrate metabolism in *L. vannamei*.

## 1. Introduction

White shrimp (*Litopenaeus vannamei*) can tolerate salinities ranging from 0.5 to 50 due to its strong ability to maintain osmosis and ion regulation, making it a widely farmed shrimp worldwide [1,2]. In 2021, freshwater aquatic animal production in China reached 665,202 t, with the freshwater production of *L. vannamei* accounting for 35% of the total production [3]. Although *L. vannamei* can be cultured on a large scale in freshwater, this factor does not mean that white shrimp can produce an optimal physiological response. Shrimp reared at low salinity can experience passive effects, including low survival (53.3%), which was observed at a salinity of 5, and increases in oxygen consumption and feed coefficients were reported [4,5]. Some research also documented that low salinity could reduce innate immune parameters [6,7], increase the toxicity of ammonia (NH_3_) and nitrite (NO_2_^−^) [8,9], decrease resistance against pathogens [7,10] and even destroy the structure of the intestinal microbiota during aquatic animal culture [11,12]. These negative effects ultimately lead to the poor aquaculture production of *L. vannamei*. Therefore, the exploitation of novel strategies to overcome the above problems is particularly important for the rapid development of the *L. vannamei* aquaculture industry. Nutritive regulation is an effective strategy to alleviate the adverse effects of low-salt stress. Previous studies have documented that dietary mineral supplements, proteins and prebiotics can improve growth performance [12,13,14,15,16] and enhance the antistress ability and immune defense ability of *L. vannamei* to alleviate the unfavorable effects of low salinity.

Prebiotics are commonly added to feed to improve the immune activity of white shrimp to environmental stress [13,17,18,19]. Among the many prebiotics, β-glucan is commonly used as an immunostimulant in the aquaculture industry [20]. β-Glucan is a complex polysaccharide present in grains, seaweeds, mushrooms, yeast and certain bacteria, distinguished by the presence of pathogen-associated molecular patterns (PAMPs) in molecules with immunomodulatory activity [21]. β-Glucan can improve the immunity and disease resistance of crustaceans, including tiger shrimp (*Penaeus monodon*) [22] and banana shrimp (*Penaeus merguiensis*) [23]. Feeding *L. vannamei* 0.2% β-glucan has positive effects on growth performance [24]. Dietary 0.05–0.20% β-glucan can improve the tolerance of Golden Pompano (*Trachinotus ovatus*) to low-salt stress [25]. Dietary β-glucans could alter the dominance of the intestinal microbiota structure in *L. vannamei* [26,27] and turbot (*Scophthalmus maximus*) [28]. Although studies have confirmed that β-glucan could promote nonspecific immune defense and improve low-salt adaptation [13], previous research involving the optimal dosage of β-glucan has not presented consistent results for *L. vannamei* under environmental stress. In addition, the effects of prebiotics on aquaculture are primarily focused on their growth and physiological and biochemical functions, but little is known about the prebiotic mechanism of prebiotics at the molecular level in shrimp under low salinity.

This study aimed to explore the optimal supplementation level of β-glucan as a prebiotic under low salinity. This study examined the effects on the growth, antioxidant capacity, immunity and intestinal microflora of *L. vannamei* under low salinity (≤3). The results of transcriptome and microbiology techniques further reveal the prebiotic mechanism of β-glucan in alleviating the unfavorable effects of low salinity.

## 2. Materials and Methods

### 2.1. Experimental Diets

The basic feed formula and nutrients are displayed in Table 1. The basic diet was formulated according to the nutritional requirements of *L. vannamei* and previous research [13]. The diet was supplemented with isonitrogenous and isolipids containing different levels (0, 0.05, 0.1, 0.2 or 0.4%) of β-glucan. Yeast (1,3)-(1,6)-β-glucan with a purity of 88% was the experimental β-glucan source. The diet material was ground with a grinder and filtered with a 60-mesh screen. All single raw materials were thoroughly mixed, and distilled water was added to make a dough, which was extruded into 2 mm diameter pellets by an extruder (CD4-1TS extruder, Guangzhou Huagongguang Electromechanical Technology Co., Ltd., Guangzhou, China). The prepared pellets were air-dried. After drying, the pellets were classified into different sizes with the most suitable sieve (12, 14, 16 and 20 mm) to ensure effective feeding and stored at −20 °C.

### 2.2. Experimental Design and Breeding

Larvae of *L. vannamei* (P5) were purchased from a commercial shrimp farm (Wenchang, China). During the acclimation period, larvae were fed a commercial diet (Alpha feed Co., Ltd., Protein 48%, Shenzhen, China) at 4% of their body weight per day. All shrimp were acclimated to a salinity of 3 at a rate of 2% reduction per day by adding fresh water. Fresh water was adequately aerated prior to adjusting the salinity of the seawater.

After finishing acclimation, shrimp (0.26 ± 0.01 g) were randomly separated into five groups: 0% β-glucan (control), 0.05% β-glucan (0.05%), 0.1% β-glucan (0.1%), 0.2% β-glucan (0.2%) and 0.4% β-glucan (0.4%), divided into five groups in 20 tanks (60 × 30 × 36 cm) with 20 shrimp, each with four replicates (October to November 2020). During the five-week experiment, daily water exchange was carried out at a rate of 1/2 the culture water volume using low-salinity (≤3) water, and residual food and feces were removed. The temperature (27 ± 2 °C), pH (7.5–8.0), total nitrogen concentration (<0.03 mg/L) and dissolved oxygen (≥7 mg/L) were maintained and checked twice a week throughout the experimental session. Shrimp were fed three times a day (7:00, 12:00 and 18:00).

### 2.3. Sampling and Growth Performance

After completing the 35-day dietary trial, all shrimp fasted for 24 h before sampling. Before sampling began, the shrimp were counted, and then they were anesthetized in an ice-water bath. The body length and body weight were randomly measured. The middle intestines and whole hepatopancreas were aseptically dissected and placed into a 1.5 mL sterile centrifuge tube, and the two tissues were prepared for the biochemical and multiomics analyses. All tube samples were immediately stored in liquid nitrogen and transferred to −80 °C for storage until further analyses. Three intestine samples were pooled into one sample per tank for microbiota analysis. Hepatopancreas samples were examined for digestive enzyme, antioxidant and immune enzyme activities. Growth-related indicators were evaluated using the following formulas:

Survival (%) = (final shrimp number/initial shrimp number) × 100

Weight gain (WG, %) = (final weight − initial weight)/initial weight × 100

Specific growth rate (SGR, % day) = 100 (Ln final weight − Ln initial weight)/number of days

### 2.4. Biochemical Assay

Eight samples of the hepatopancreas (1 g) from two shrimp per tank per treatment were accurately weighed, transferred to sterile centrifuge tubes and homogenized in a cold 0.86 saline solution (1:9, *w*/*v*) using a tissue grinder (Shanghai Jingxin, Shanghai, China) (60 Hz, 30 s) and centrifuged at 1500× *g* (4 °C/15 min, 3–18 KS, Sigma, Osterode, Germany). After centrifugation, the supernatant was collected to determine the total protein concentration, digestive enzymes (protease, amylase and lipase), total antioxidant capacity (T-AOC), catalase (CAT), malondialdehyde content (MDA), superoxide dismutase (SOD), acid phosphatase (ACP) and alkaline phosphatase (AKP). These biochemical enzyme activities in the hepatopancreas were measured according to procedures described in [29,30]. All hepatopancreatic biochemical indices were determined using commercial assay kits (Nanjing Jiancheng Bioengineering Institute, Nanjing, China), and the operating procedures were used according to the manufacturer’s instructions.

### 2.5. Intestinal Microbiota Analysis

Total genomic DNA was extracted from intestinal samples using the TAB/SDS method. DNA quality was measured using a NanoDrop spectrophotometer (Thermo, Wilmington, DE, USA). Generation sequencing was performed on an Illumina HiSeq system according to the protocol of Majorbio Biopharm Technology Co., Ltd. (Shanghai, China). The hypervariable V3–V4 region of the 16S rRNA gene was amplified by using primers 338 (forward 5-ACTCCTACGGGAGGCAGCA-3) and 806 (reverse 5-GGACTACHVGGGTWTCTAAT-3). A TruSeq^®^ DNA PCR-free Sample Preparation Kit (Illumina, CA, USA) was used to construct the library, and index codes were added. Finally, the libraries were carried out on an Illumina HiSeq 2500 platform. All statistical analyses of the results were performed using the R package software (version 2.11 http://sourceforge.net/projects/rdp-classifier/; accessed on 10 January 2021). Alpha-diversity indices (ACE, Chao1, Simpson, Shannon and Observed_species) were determined using QIIME software (http://www.mothur.org/wiki/Calculators; accessed on 10 January 2021), and statistical analyses were performed using one-way analysis of variance (ANOVA) (SPSS 23.0). The raw sequences of intestinal microbiota in this study were submitted to Sequence ReadArchive (SRA) under GenBank database number PRJNA842883.

### 2.6. Transcriptomic Analysis

Total RNA was extracted from the hepatopancreas of individual shrimp (three samples per treatment) using TRIzol Reagent (Ambion, CA, USA). The quality and concentration of the RNA were determined using a 1% agarose gel on a 2100 Bioanalyzer (Agilent Technologies, Palo Alto, CA, USA) and ND-2000 (Thermo, Wilmington, DE, USA). One microgram of total RNA per sample was used to construct a sequencing library using a TruSeq RNA sample preparation kit from Illumina (San Diego, CA, USA). The prepared cDNA libraries were sequenced on the Illumina HiSeq™ 4000 platform at Majorbio Bio-Pharm Technology Co., Ltd. (Shanghai, China). Raw paired-end reads were quality-checked with default parameters. Clean reads were individually screened using Trimmomatic software (http://www.usadellab.org/cms/uploads/supplementary/Trimmomatic; accessed on 13 January 2021). Further, total clean reads were separately aligned to the reference *L. vannamei* genome in orientation mode using TopHat (http://tophat.cbcb.umd.edu; accessed on 13 January 2021). The expression levels of unigenes were calculated according to the fragments per kilobase of transcripts per million reads (TPM) to identify differentially expressed genes (DEGs) between different samples. RSEM was used to estimate gene abundances (http://deweylab.biostat.wisc.edu/rsem/; accessed on 10 January 2021). Essentially, differential expression analysis was performed using DESeq2/DEGseq/EdgeR with a Q value of 0.05. DEGs with |log2FC| > 1 and Q value ≤ 0.05 (DESeq2 or EdgeR)/Q value ≤ 0.001 (DEGseq) were considered DEGs. Kyoto Encyclopedia of Genes and Genomics (KEGG) pathway analysis of the DEGs was performed using KOBAS software (http://kobas.cbi.pku.edu.cn/home.do; accessed on 10 January 2021). In addition, KEGG enrichment analyses were conducted with Bonferroni-corrected *p* values < 0.05. A false discovery rate < 0.01 and FC > 2 were considered for the significance analysis. The RNA-seq data were deposited in the NCBI SRA database (PRJNA846923).

### 2.7. Statistical Analysis

To clarify the mechanism of dietary β-glucan on growth, biochemical and omics analyses were performed. SPSS 23 software (IBM, New York, NY, USA) was used to carry out all statistical analyses. The data are expressed as the mean ± standard error (mean ± SE). One-way ANOVA was chosen to determine the significant effects among different groups, followed by multiple comparisons by Duncan’s multiple comparison test. The statistical significance level was set at *p* < 0.05. In addition, to determine whether the effect was quadratic, a follow-up trend analysis using the method of orthogonal polynomial contrasts was performed using SPSS 23. The assumptions of normality and homoscedasticity were confirmed before conducting any statistical analyses. The relationship between WG, SGR and dietary β-glucan was estimated through second-order polynomial regression [31], and the optimal dietary β-glucan requirement was also evaluated.

## 3. Results

### 3.1. Growth Performance

**Figure 1:**
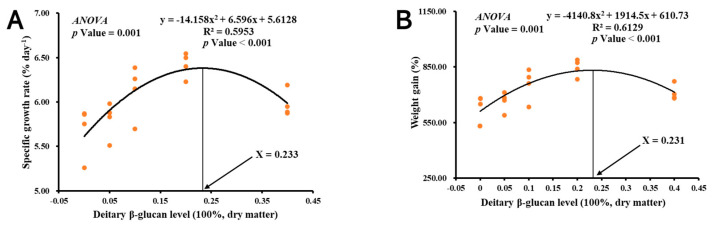
Relationships of (**A**) specific growth rate and (**B**) weight gain with dietary β-glucan levels based on second−order polynomial regression analysis of Juvenile *L. vannamei*, where Xopt represents the dietary β-glucan level (*n* = 4).

The growth performance results of the 35-day trial are shown in Table 2. There was no significant difference in survival among the groups (*p* > 0.05). The growth performance of shrimp was the highest in the 0.2% group, which was significantly higher than that of the other groups (*p* < 0.05) but not significantly different from that of the 0.1% group (*p* > 0.05). Furthermore, a second-order polynomial regression analysis of WG and SGR against dietary β-glucan indicated that the ideal dietary β-glucan level was 0.2–0.23% of dry matter (Figure 1).

### 3.2. Hepatopancreatic Digestive Enzyme Activity

**Figure 2:**
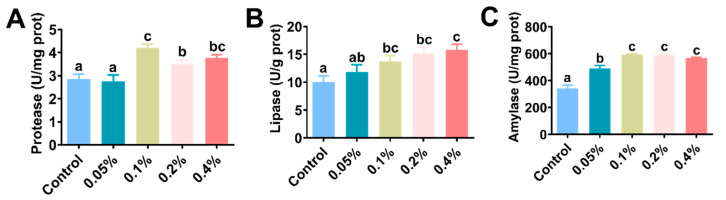
Effects of different doses of β-glucan on the (**A**) protease, (**B**) lipase and (**C**) amylase enzyme activities in Juvenile *L. vannamei*. Different letters indicate significant differences (*p* < 0.05) among groups. All data are expressed as the mean ± SE (*n* = 4).

The hepatopancreatic digestive enzyme activities are shown in Figure 2 and Table 3. Hepatopancreas protease and lipase activities were significantly higher in the 0.1%, 0.2% and 0.4% groups than in the control group (*p* < 0.05) (Figure 2A,B). The hepatopancreas amylase activity was also significantly increased with the addition of β-glucan compared with the control group (*p* < 0.05) (Figure 2C).

### 3.3. Antioxidant Capacity and Immunity

**Figure 3:**
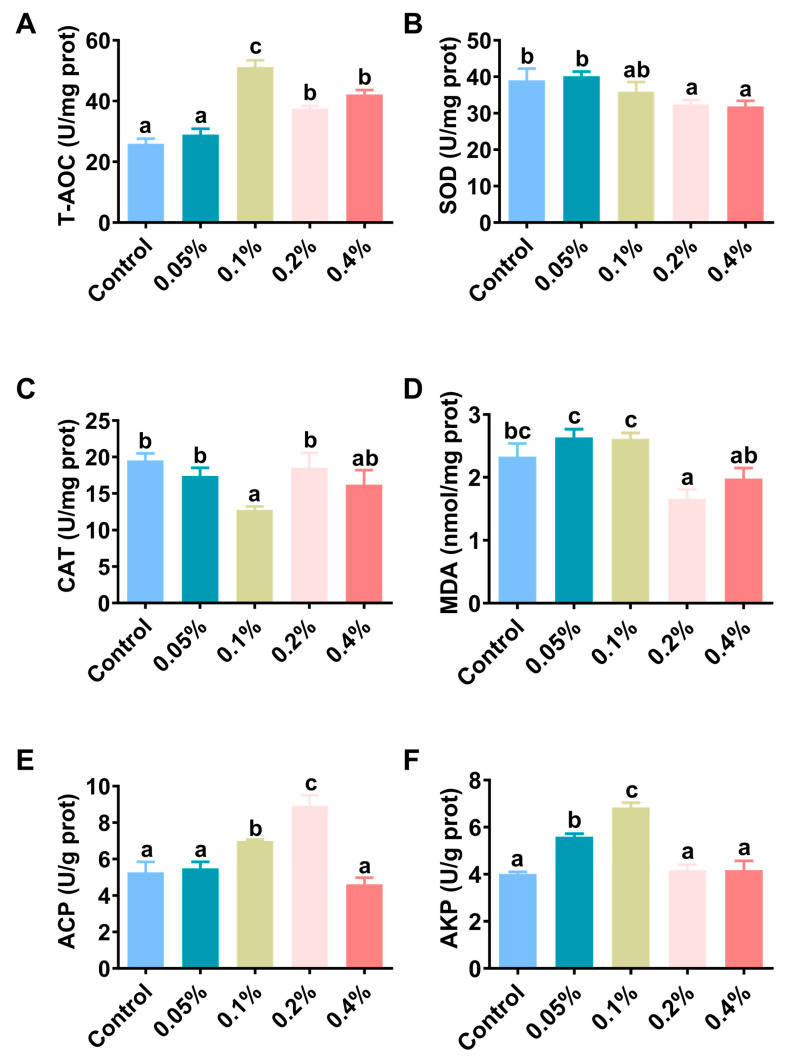
Effects of different doses of β-glucan on the (**A**) total antioxidant capacity, (**B**) superoxide dismutase, (**C**) catalase, (**D**) malondialdehyde content, (**E**) acid phosphatase and (**F**) alkaline phosphatase of Juvenile *L. vannamei*. Different letters indicate significant differences (*p* < 0.05) among groups. All data are expressed as the mean ± SE (*n* = 4).

The hepatopancreatic antioxidant performance and nonspecific immune response results are shown in Figure 3 and Table 3. The T-AOC enzyme activity in the hepatopancreas of shrimp in the 0.1%, 0.2% and 0.4% groups was significantly higher than that in the control group (*p* < 0.05) (Figure 3A). The SOD enzyme activity in the hepatopancreas of shrimp in the 0.1% group was significantly higher than that in the other groups (*p* < 0.05) (Figure 3B). The CAT enzyme activity in the hepatopancreas of shrimp in the 0.1% group was significantly lower than that in the other groups (*p* < 0.05) but not significantly different from that in the 0.4% group (*p* > 0.05) (Figure 3C). The MDA content of the hepatopancreas in shrimp in the 0.2% group was significantly increased compared with that in shrimp in the other groups (*p* < 0.05) but was not significantly different from that in shrimp in the 0.4% group (*p* > 0.05) (Figure 3D). The ACP enzyme activity in the hepatopancreas of shrimp in the 0.1% and 0.2% groups was significantly higher than that in the other groups (*p* < 0.05) (Figure 3E). The AKP enzyme activity in the hepatopancreas of shrimp in the 0.05% and 0.1% groups was significantly higher than that in the other groups (*p* < 0.05) (Figure 3F).

### 3.4. Intestinal Microbiota Analysis

**Figure 4:**
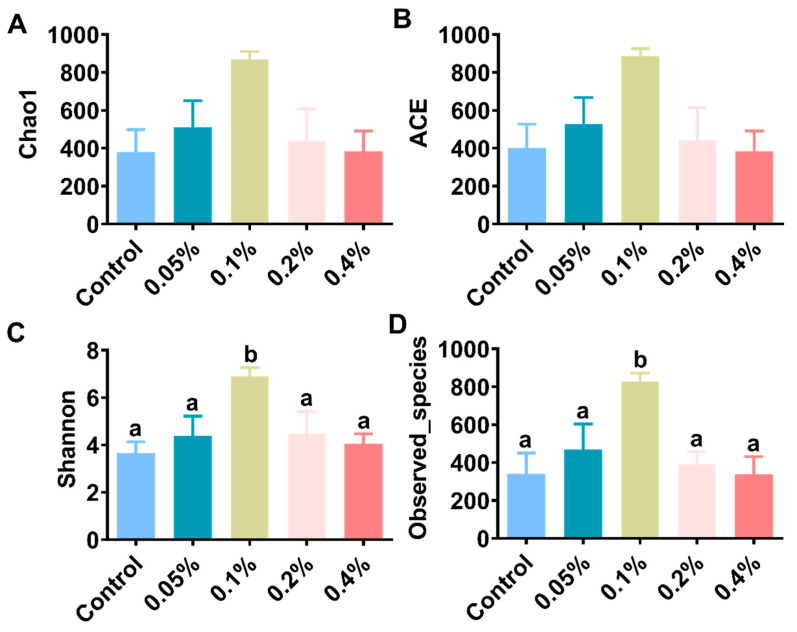
Effects of different doses of β-glucan on the alpha-diversity using (**A**) Chao1 estimator, (**B**) ACE estimator, (**C**) Shannon estimator and (**D**) observed species in gut microbiota in Juvenile *L. vannamei*. Different letters indicate significant differences (*p* < 0.05) among groups. All data are expressed as the mean ± SE (*n* = 4).

**Figure 5:**
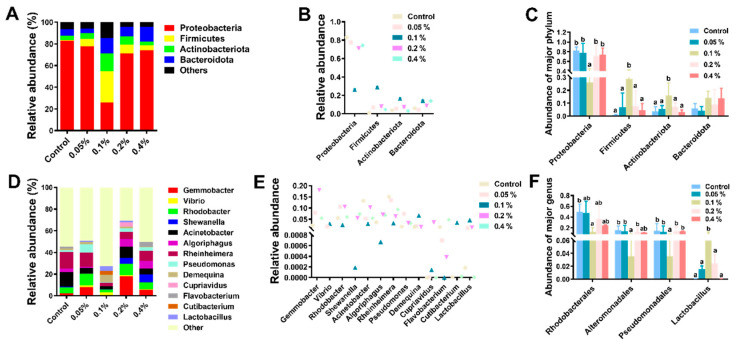
Effects of different doses of β-glucan on the gut microbiota composition of Juvenile *L. vannamei* at the phylum and genus levels. (**A**) Microbiota composition at the phylum level with the relative abundance of the top four. (**B**) Relative abundance of gut microbiota at the phylum level. (**C**) Differences in the relative abundance of phylum taxa among groups. (**D**) Microbiota composition at the genus level with the relative abundance of the top thirteen. (**E**) Relative abundance of gut microbiota at the genus level. (**F**) Differences in the relative abundance of genus taxa among groups. Different letters indicate significant differences (*p* < 0.05) among groups. All data are expressed as the mean ± SE (*n* = 4).

**Figure 6:**
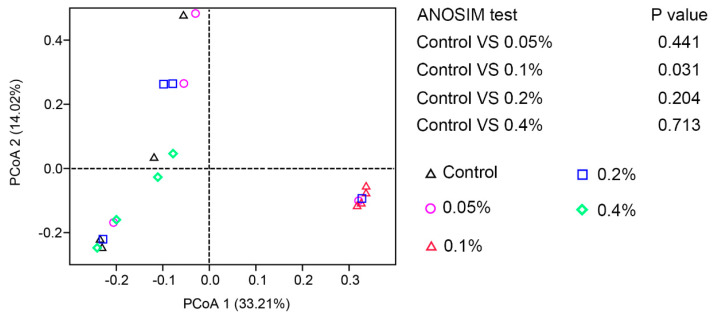
Effects of different doses of β-glucan on the β−diversity of gut microbiota in Juvenile *L. vannamei*. PCoA of the microbiota at the OTU level based on Jaccard distances. Analysis of similarity (ANOSIM) was performed to evaluate the overall differences in bacterial community structure based on Jaccard distance (*n* = 4).

In this study, the analysis of the intestinal microflora showed that a total of 2,384,213 high-quality sequences were acquired in all samples. Each sample had an average of 99,342 sequences. The observed species ranged from 338.00 to 828.25. Supplementation with 0.1% β-glucan significantly increased the alpha-diversity indices (Shannon and Observed_species) compared with those of the other groups (*p* < 0.05), as shown in Figure 4 and Table S1. At the phylum level, the most dominant phylum was Proteobacteria, followed by Firmicutes, Actinobacteriota and Bacteroidota in all samples (Figure 5A,B), and Firmicutes and Actinobacteriota were dominant in the 0.1% group (Figure 5C and Table S1). Supplementation with 0.1% significantly induced an increase in the relative abundance of intestinal microbial species compared with those of the other groups of *L. vannamei* (*p* < 0.05). At the genus level, the relative abundances of Vibrio, Rheinheimera and Demequina in the 0.1% group were significantly lower than those in the other groups, but the relative abundance of Lacrobacillus in the 0.1% group was significantly higher than those in the other groups (Figure 5D–F and Table S1). In addition, the principal coordinate analysis (PCoA) cluster analysis indicated that the overall structure of the intestinal microflora in the 0.1% group was significantly different compared with the control group (Figure 6).

### 3.5. Transcriptome Analysis

**Figure 7:**
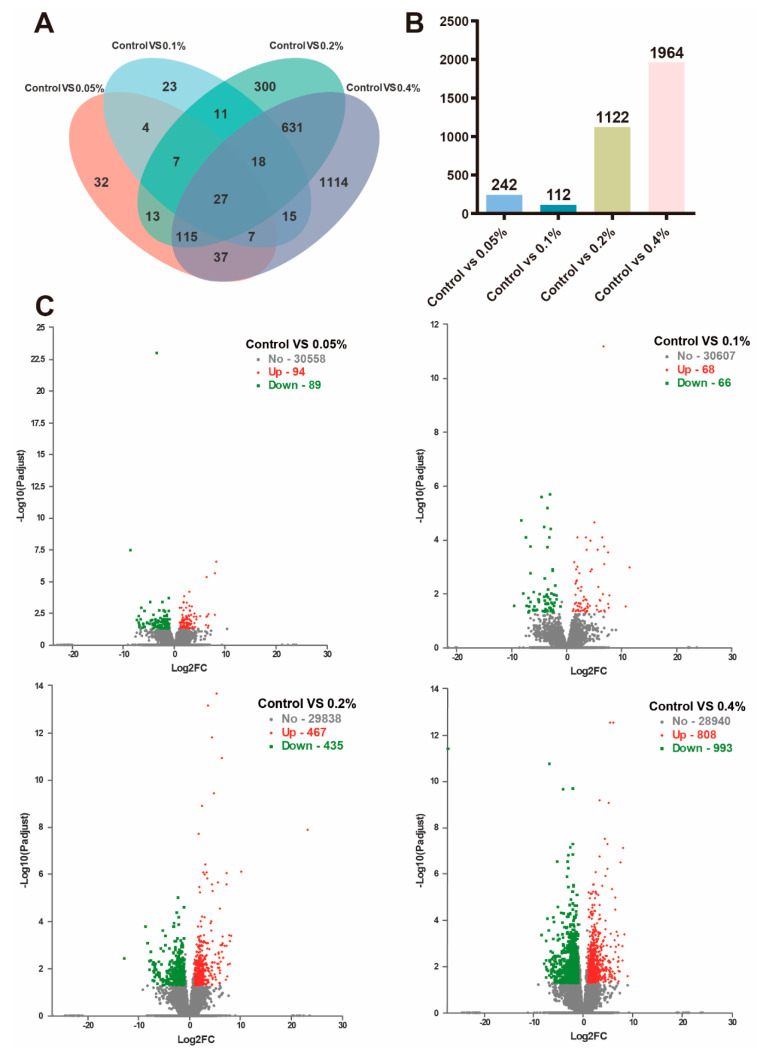
Changes in transcriptome levels of differentially expressed genes with different doses of β-glucan in Juvenile *L. vannamei*. (**A**,**B**) The Venn diagram shows the number of common or unique differential genes among groups (0.05%, 0.1%, 0.2% and 0.4%) compared with the control group. (**C**) Volcano map showing the number of upregulated and downregulated genes in the control vs. 0.05%, control vs. 0.1%, control vs. 0.2% and control vs. 0.4% groups; the *X*-axis is represented by log2 (fold change), and the larger the variation, the wider the distribution; the *Y*-axis is represented by −log10 (Padj), a negative logarithm of the adjusted *p*-value; the green dots represent downregulated genes, the red dots represent upregulated genes, and the gray dots represent genes that do not differ significantly (*n* = 4).

Among the five treatment groups, 2354 differentially expressed genes (DEGs) were identified. As shown in Figure 7, a total of 27 DEGs were shared among the treatment groups, while 32, 23, 300 and 1114 DEGs were significantly expressed only in the control vs. 0.05% group, control vs. 0.1% group, control vs. 0.2% group and control vs. 0.4% group, respectively (Figure 7A,B). A total of 183 DEGs were found in the control vs. 0.05% group, including 94 upregulated and 89 downregulated genes; 134 DEGs were found in the control vs. 0.1% group, including 68 upregulated and 66 downregulated genes; 902 DEGs were found in the control vs. 0.2% group, including 467 upregulated and 435 downregulated genes; and 1801 DEGs were found in the control vs. 0.4% group, including 808 upregulated and 993 downregulated genes (Figure 7C). Compared with the control group, there were 27 differentially expressed genes treated with different doses of β-glucan. Specific information on these 27 common DEGs is shown in Table S2. Significant DEGs were studied using KEGG pathway enrichment statistics to study the important functional pathways of DEGs (Table S3). The phenoloxidase gene, antimicrobial peptide gene and Na^+^/K^+^/2Cl^−^ cotransporter gene expression levels were significantly upregulated in the β-glucan addition group. The KEGG analyses of DEGs showed that dietary β-glucan under low salinity also regulated signal transduction pathways (RIG-I-like receptor, PPAR, JAK-STAT, prolactin, neurotrophic factor, AMPK, Toll-like receptor, TNF and sphingolipid) and activated KEGG pathways associated with carbohydrate metabolism (starch and sucrose metabolism, carbohydrate digestion and absorption and galactose metabolism).

## 4. Discussion

*L. vannamei* can adapt to extensive environmental salinity fluctuations; its optimal growth rate occurs at ≥20 salinity [11], and it faces hyposaline stress under low salinity [32]. Various prebiotics have been proven to alleviate hyposaline stress; for instance, inulin can cope with low-salinity stress in shrimp [19]. Dietary β-glucan significantly increased the WG, SGR and PO activity of Pacific white shrimp [27] and post-larval *P. monodon* [33]. This study revealed that 0.1% and 0.2% β-glucan increased the growth performance, protease activity, lipase activity and amylase activity of *L. vannamei* under low-salinity stress. β-Glucan could improve the growth of *L. vannamei*, which could be due to the increased digestive capacity and the absorption and accumulation of nutrients. As with almost all functional substances, β-glucan is often underutilized in practical farming due to a lack of scientific strategies for its use, but this study showed that β-glucan improved growth performance at low salinity, suggesting that β-glucan has superior production applications. Similar results showed that the addition of 0.02% β-glucan (0.04%) increased the weight gain of *L. vannamei* [13]. Prebiotic xylooligosaccharides fed to crucian carp (*Carassius auratus*) caused an increase in digestive enzymes [34], while mannanoligosaccharides significantly enhanced digestive enzymes in *Pangasianodon hypophthalmus* [35]. Multiple stressors, such as low salinity, nitrite and ammonia, can produce excess reactive oxygen species (ROS) that damage the organism [36,37]. SOD and CAT enzymes, as antioxidant enzymes, can remove excess ROS, and GPx also has an efficient antioxidant capacity [38]. This study demonstrated that the antioxidant enzyme activity was increased due to supplementation with β-glucan compared with the control. Similarly, this research showed that 0.02% and 0.04% β-glucan could have a positive effect on SOD and GPX enzyme activities in *L. vannamei* [13]. Dietary β-glucan enhanced the SOD activity in *Penaeus monodon* [27,39], and similar results were found with the addition of inulin in Pacific white shrimp [19]. These results suggest that dietary β-glucan could reduce oxidative damage caused by low-salinity stress by increasing the activities of antioxidant enzymes. In addition, this experiment showed that 0.1% β-glucan helped to increase the ACP and AKP activities in *L. vannamei*. ACP and AKP are capable of assisting, modulating and accelerating phagocytosis [40]. A previous study also showed that the oral administration of β-glucan enhanced ACP activity [41]. In this study, the significant activation of SOD, ACP and AKP activities clearly illustrates that β-glucan can be used as an immunostimulatory agent in Pacific white shrimp, which is beneficial for improving the immunity of shrimp in a low-salinity environment.

The effects of prebiotics are closely related to the dosage in aquatic animals [17,18,19]. In this study, the growth performance of *L. vannamei* increased with the additive dose (0–0.2%) but started to decrease when β-glucan was 0.4%. This indicated that there was no linear relationship between the growth-promoting effect of prebiotics on *L. vannamei* and the additive dose. In addition to the growth performance indicators, other indicators, such as digestive enzyme activity, total antioxidant capacity, malondialdehyde content, AKP, ACP activity and so on, all showed the same phenomenon. A previous study found that the addition of 0.064% β-glucan improved the weight gain of *L. vannamei* [42]. Moreover, 0.02% dietary β-glucan showed higher levels of immune gene expression in *L. vannamei* [13]. In this study, based on the growth performance, oxidation resistance and immune indices, it was speculated that the optimal β-glucan additive level in the diet of *L. vannamei* in low-salinity culture conditions was 0.1%. It should be noted that the most appropriate prebiotic dose may vary depending on the species of aquatic animal, the type of prebiotic, the structure and dietary conditions, so there is no simple linear relationship.

The intestine not only plays a vital role in nutrient absorption and metabolic activity but also improves environmental stress and provides a defense against pathogens [43]. Intestinal microorganisms and their metabolites can directly or indirectly affect the physiological functions of animals, including metabolism and immunity [44]. Recently, most research on Pacific white shrimp has paid more attention to the relationships between shrimp health and intestinal microbiota [45,46]. Dietary prebiotics can modulate the microbial population in the intestine of the host [47]. This study showed that adding 0.1% β-glucan significantly increased the intestinal microflora values of α-diversity indices. The effect of prebiotics on the α-diversity of intestinal flora in aquatic animals is still controversial. Some studies have shown that inulin and β-glucan have no effect on the α-diversity of intestinal flora in white shrimp [13,19]. However, some studies have documented that prebiotics increase the α-diversity of intestinal flora [48]. Some results suggested that prebiotics enhanced immunity in the host by stimulating the growth of probiotics such as *Lactobacillus* and *Bacillus* [49,50]. This study showed that the dominant phylum and genus in the intestine were significantly changed by the addition of β-glucan. The addition of 0.1% β-glucan could affect the bacterial community in the intestine of *L. vannamei* by increasing the relative abundance of *Lactobacillus*, thereby maintaining a more stable intestinal environment and resisting the invasion of pathogens [45]. The vast majority of *Lactobacillus* species are considered probiotics for aquatic animals [51]. Therefore, dietary β-glucan could have beneficial effects by promoting the occupancy of these intestinal probiotics in the host. Furthermore, we hypothesize that the practical value of β-glucan in aquaculture is mainly through intestinal microbiota and physiological changes that synergistically improve the physiological health of farmed shrimp.

Transcriptome sequencing technology is a significant approach for quantifying transcriptional expression in nonmodel species [52,53]. This strategy has been used in the environmental stress response, such as different salinities [54] and temperatures in *L. vannamei* [55]. In this study, differential gene expression analysis revealed that there were 27 common differential gene changes between the β-glucan groups at different doses and the control group, and these differential gene changes ranged from 1 to 3. The number of upregulated DEGs was much larger than that of downregulated DEGs. This result indicated that β-glucan alleviated low-salinity stress mainly by activating gene expression levels in *L. vannamei*. The expression levels of phenoloxidase genes and antimicrobial peptide genes were significantly upregulated with β-glucan addition. Phenol oxidase activity has a positive correlation with disease [56]. Shrimp encode and produce antimicrobial peptides (AMPs) as part of their innate immune response [57]. These results suggest that dietary β-glucan could enhance the nonspecific immune function of white shrimp under low salinity by enhancing the expression of immune-related genes, thus producing a probiotic effect on the host. In addition, compared with the control group, the expression level of the Na^+^/K^+^/2Cl^−^ cotransporter gene was significantly upregulated in the β-glucan group at different doses and was closely related to osmotic pressure regulation [58]. Similar results were found in a previous study, where prebiotic inulin significantly upregulated metabolic pathways related to osmotic pressure regulation (aldosterone-mediated sodium reuptake metabolic pathway) in *L. vannamei* under low-salinity stress [19]. These results suggest that the prebiotic effect of prebiotics on the host may be mediated by regulating osmotic pressure at low salinity. However, there is limited information on the effect of prebiotics on osmotic regulation, and more research is needed. In the present study, transcriptome analyses were performed in *L. vannamei* receiving dietary β-glucan to investigate how β-glucan plays an active role. KEGG analyses of DEGs showed that β-glucan has significant effects on pathways related to energy metabolism and immune defense. Low-salinity stress may affect the metabolism and immune system of *L. vannamei*. It was speculated that β-glucan could exert its probiotic effect on the host by regulating these signaling pathways. Osmotic regulation requires a certain amount of energy in crustaceans to ensure normal metabolism [2]. Many enzymes and transporters are relevant to iono- and osmoregulatory processes, which consume large amounts of energy [59]. In this study, KEGG pathways related to carbohydrate metabolism, such as starch and sucrose metabolism, carbohydrate digestion and absorption, and galactose metabolism, were significantly enriched under low salinity by adding β-glucan. To date, dietary supplementation with xylooligosaccharides (XOS) has been demonstrated to be effective in improving blood glucose [60]. A previous study showed that the prebiotic inulin enriches different metabolic pathways, including glycolysis and gluconeogenesis [61], consistent with this study. These results indicate that dietary β-glucan addition can improve immune defense and energy metabolism disorders under low-salinity stress. Furthermore, the prebiotic β-glucan could meet energy requirements under low salinity by activating KEGG pathways related to carbohydrate metabolism, thus producing a probiotic effect on the host.

## 5. Conclusions

The addition of β-glucan could improve the antioxidant capacity of *L. vannamei* by enhancing the activities of T-AOC, SOD and CAT to relieve low-salinity stress (≤3). In addition, dietary β-glucan addition could enhance growth performance and effectively alleviate the physiological effects by enhancing biochemical indicators (digestive and nonspecific immune activity) and improving intestinal microbiota and carbohydrate metabolism (starch and sucrose metabolism, carbohydrate digestion and absorption, galactose metabolism, etc.). In addition, it is recommended that the β-glucan dose added to the diet be 0.1–0.2% to improve growth performance and relieve low-salinity stress (≤3). These results can provide a reference for the practical application value and farming costs of β-glucan in aquaculture, which will set a model for subsequent research on functional substances in *L. vannamei* and contribute to the green, healthy and sustainable development of the global aquaculture industry.

## Figures and Tables

**Table 1:** Dietary formulations and proximate composition of the experimental diets (% dry matter).

Ingredients (%)	Dietary β-Glucan Concentrations				
	0	0.05%	0.1%	0.2%	0.4%
Fish meal	26	26	26	26	26
Soybean meal	28	28	28	28	28
Com starch	23	23	23	23	23
Shrimp meal	4	4	4	4	4
Calcium dihydrogen phosphate	1.5	1.5	1.5	1.5	1.5
Vitamin premix ^a^	2	2	2	2	2
Mineral premix ^b^	2	2	2	2	2
Choline chloride	1	1	1	1	1
Fish oil	2.5	2.5	2.5	2.5	2.5
Soybean oil	2.5	2.5	2.5	2.5	2.5
Soybean lecithin	1	1	1	1	1
Cholesterol	0.5	0.5	0.5	0.5	0.5
Carboxymethylcellulose (CMC)	3	3	3	3	3
Butylated hydroxytoluene (BHT)	0.1	0.1	0.1	0.1	0.1
Microcrystalline cellulose	2.9	2.85	2.8	2.7	2.5
β-1,3-Glucan ^c^	0	0.05	0.1	0.2	0. 4
Total	100	100	100	100	100
Nutrient levels (%)					
Crude protein	35.2	35.4	35.5	35.5	35.4
Crude lipid	7.6	7.6	7.6	7.6	7.6
Ash	10.3	10.4	10.7	10.7	10.6
Moisture	9.2	9.2	9.2	9.2	9.3

^a^ Vitamin premix (g/kg premix): vitamin A acetate (500,000 IU/g), 0.960; L-ascorbyl-2-polyphosphate 35% Active C, 71.420; folic acid, 0.360; biotin, 5.000; riboflavin, 6.000; DL Ca-pantothenate, 10.000; pyridoxine HCl, 2.000; 1% vitamin B12, 0.400; thiamin HCl, 1.000; Menadione, 4.000; DL-alpha-tocopheryl acetate (250 IU/g), 16.000; nicotinic acid, 10.000; vitamin D (500,000 IU/g), 1.600; defatted rice bran, 871.260. ^b^ Mineral premix (g/kg premix): zinc sulfate monohydrate, 20.585; calcium iodate, 0.117; cupric sulfate pentahydrate, 0.625; manganous sulfate monohydrate, 1.625; magnesium sulfate monohydrate, 39.860; cobalt chloride, 0.010; ferrous sulfate monohydrate, 11.179; sodium selenite, 0.025; calcium hydrogen phosphate dihydrate, 166.442; defatted rice bran, 759.532. ^c^ β-Glucan was purchased from Xi’an Ruilin Biotechnology Co., Ltd., Xi’an, China.

**Table 2:** Regression analysis of the growth performance of Juvenile *L. vannamei* fed different dietary β-glucan levels for 5 weeks.

Dietary β-Glucan Levels (%) ^a^	IW (g)	FW (g)	WG (%)	SGR (% Day^−1^)	Survival (%)
0	0.27	1.91 ± 0.07 ^a^	634.08 ± 35.42 ^a^	5.68 ± 0.14 ^a^	90 ± 4.56
0.05	0.27	2.01 ± 0.09 ^a^	663.15 ± 26.37 ^ab^	5.80 ± 0.10 ^ab^	90 ± 2.04
0.1	0.26	2.23 ± 0.09 ^b^	756.20 ± 43.09 ^bc^	6.12 ± 0.15 ^bc^	95 ± 3.54
0.2	0.25	2.36 ± 0.04 ^b^	845.86 ± 22.82 ^c^	6.42 ± 0.07 ^c^	90 ± 1.44
0.4	0.25	1.92 ± 0.02 ^a^	710.36 ± 21.32 ^ab^	5.98 ± 0.07 ^ab^	92.5 ± 1.25
ANOVA (*p* value)	0.533	0.001	0.002	0.003	0.444
SOP (*n* = 4)					
Adj. R^2^		0.6423	0.5365	0.0257	
*p* Value		<0.001	<0.001	<0.001	

Note: ^a^ Values represent the means of four replicate tanks (*n* = 4). Different superscript lowercase letters within a column indicate significant differences (*p* < 0.05). IW, initial weight; FW, final weight; WG, weight gain; SGR, specific growth rate; SOP, second-order polynomial; Adj. R^2^, adjusted R-squared.

**Table 3:** Regression analysis of biochemical assays in the hepatopancreas of Juvenile *L. vannamei* fed different dietary β-glucan levels.

Enzyme ^a^	Regression Analysis (*n* = 4) ^b^		Enzyme	Regression Analysis (*n* = 4)	
	SOP			SOP	
	Adj. R^2^	*p* Value		Adj. R^2^	*p* Value
T-AOC	0.3935	0.046	Protease	0.326	0.035
SOD	0.3979	0.695	Lipase	0.5556	0.002
CAT	0.056	0.567	Amylase	0.8093	0.005
MDA	0.2971	0.023			
ACP	0.7077	0.765			
AKP	0.2194	0.193			

Note: ^a^ T-AOC, total antioxidant capacity; SOD, superoxide dismutase; CAT, catalase; MDA, malondialdehyde content; ACP, acid phosphatase; AKP, alkaline phosphatase. ^b^ SOP, second-order polynomial; Adj. R^2^, adjusted R-squared.

## Data Availability

The data provided in this study have been uploaded to the NCBI database. The accession numbers are PRJNA842883 and PRJNA846923, and the links are https://www.ncbi.nlm.nih.gov/sra/PRJNA842883 (accessed on 23 May 2022) and https://submit.ncbi.nlm.nih.gov/subs/PRJN-A846923 (accessed on 23 May 2022).

## Supplementary Materials

The following supporting information can be downloaded at: https://www.mdpi.com/article/10.3390/antiox11112282/s1. Table S1: Regression analysis of the gut microbiota composition in *L. vannamei* fed different dietary β-glucan levels; Table S2: Shared differentially expressed genes between the control, 0.05%, 0.1%, 0.2% and 0.4% groups; Table S3: KEGG pathway enrichment of the differentially expressed genes between control, 0.05%, 0.1%, 0.2% and 0.4%.
