# Supplementary material for: Effects of Different Dietary β-Glucan Levels on Antioxidant Capacity and Immunity, Gut Microbiota and Transcriptome Responses of White Shrimp (Litopenaeus vannamei) under Low Salinity
Source: Antioxidants (Basel). 2022 Nov 18;11(11):2282. doi: 10.3390/antiox11112282 (PMC9686864; doi:10.3390/antiox11112282)
Supplement: Supplementary file 1 [file antioxidants-11-02282-s001.zip › antioxidants-2015630-supplementary.pdf]

Table S1. Regression analysis of the gut microbiota composition of *L. vannamei* fed different dietary β-glucan levels

**Alpha diversity**	**Regression analysis (n = 4)** [a]		**phylum level**	**Regression analysis (n = 4)**		**genus level**	**Regression analysis (n = 4)**	
	**SOP**			**SOP**			**SOP**	
	**Adj. R^2**	***p* Value**		**Adj. R^2**	***p* Value**		**Adj. R^2**	***p* Value**
Chao1	0.126	0.318	Proteobacteria	0.1678	0.21	Rhodobacterales	0.1752	0.194
ACE	0.1279	0.312	Firmicutes	0.1869	0.172	Alteromonadales	0.087	0.458
Shannon	0.168	0.209	Actinobacteriota	0.2515	0.085	Pseudomonadales	0.047	0.664
Observed_ species	0.1637	0.219	Bacteroidota	0.1487	0.254	Lactobacillus	0.3028	0.047

Note: [a] SOP, second-order polynomial; Adj. R^2, adjusted R square.

Table S2. Shared differentially expressed genes between the control, 0.05%, 0.1%, 0.2% and 0.4% groups

Gene ID	NR Database comments	0.05%/Control		0.1%/Control		0.2%/Control		0.4%/Control	
		Log2FoldChange	Regulated	Log2FoldChange	Regulated	Log2FoldChange	Regulated	Log2FoldChange	Regulated
TRINITY_DN13281_c0_g1	phenoloxidase 3-like	2.59	UP	4.31	UP	4.50	UP	5.17	UP
TRINITY_DN1333_c0_g1	Unknown	-1.38	DOWN	-1.30	DOWN	-1.77	DOWN	-1.81	DOWN
TRINITY_DN1375_c0_g1	hemocyte transglutaminase	2.63	UP	2.50	UP	3.68	UP	2.80	UP
TRINITY_DN14962_c0_g1	BACE 1 [Penaeus vannamei]	1.44	UP	1.80	UP	1.92	UP	1.80	UP
TRINITY_DN16841_c0_g1	putative trehalase isoform X1	2.11	UP	2.12	UP	3.02	UP	2.76	UP
TRINITY_DN18662_c0_g1	C-Maf-inducing protein, partial	1.24	UP	1.30	UP	2.01	UP	1.83	UP
TRINITY_DN1932_c0_g1	Na+/K+/2Cl- cotransporter [Penaeus monodon]	1.07	UP	1.69	UP	1.39	UP	1.84	UP
TRINITY_DN22420_c0_g1	uncharacterized protein LOC113811243	3.99	UP	4.23	UP	5.58	UP	6.13	UP
TRINITY_DN2282_c0_g1	Unknown	-2.49	DOWN	-2.94	DOWN	-2.13	DOWN	-2.68	DOWN
TRINITY_DN23062_c0_g6	uncharacterized protein LOC113802105	1.62	UP	1.54	UP	1.99	UP	1.97	UP
TRINITY_DN2608_c2_g2	Unknown	-1.01	DOWN	-1.09	DOWN	-1.34	DOWN	-1.51	DOWN
TRINITY_DN2701_c0_g1	protein FAM57A-like	2.45	UP	2.04	UP	2.06	UP	2.60	UP
TRINITY_DN2958_c0_g1	Unknown	2.89	UP	3.87	UP	4.27	UP	4.06	UP
TRINITY_DN3380_c0_g2	retinoid-inducible serine carboxypeptidase-like	2.32	UP	2.51	UP	2.50	UP	2.61	UP
TRINITY_DN3720_c0_g1	basic proline-rich protein-like isoform X1	2.01	UP	2.02	UP	2.43	UP	2.03	UP
TRINITY_DN3972_c0_g1	carbohydrate sulfotransferase 9-like	-2.54	DOWN	-2.10	DOWN	-1.84	DOWN	-1.64	DOWN
TRINITY_DN4411_c0_g1	nose resistant to fluoxetine protein 6-like	2.60	UP	2.95	UP	2.80	UP	3.83	UP
TRINITY_DN5511_c0_g1	putative antimicrobial peptide	3.53	UP	5.13	UP	5.36	UP	5.55	UP
TRINITY_DN5673_c0_g1	VICP1	2.81	UP	3.68	UP	4.20	UP	4.24	UP
TRINITY_DN567_c0_g1	late cornified envelope-like proline-rich protein 1	1.93	UP	2.69	UP	3.41	UP	3.31	UP
TRINITY_DN5711_c0_g1	Unknown	-2.90	DOWN	-4.15	DOWN	-2.41	DOWN	-1.46	DOWN
TRINITY_DN581_c0_g1	hypothetical protein C7M84_021358	2.31	UP	2.31	UP	1.98	UP	2.12	UP

TRINITY_DN6139_c0_g1	Unknown	-1.75	DOWN	-1.84	DOWN	-1.91	DOWN	-2.44	DOWN
TRINITY_DN7521_c0_g1	serine/threonine-protein phosphatase 6 regulatory ankyrin repeat subunit B-like	-4.12	DOWN	-5.00	DOWN	-3.63	DOWN	-5.35	DOWN
TRINITY_DN7883_c0_g1	protein kibra-like	2.15	UP	2.24	UP	2.26	UP	2.73	UP
TRINITY_DN8599_c0_g1	T-box transcription factor TBX18-like	1.70	UP	1.60	UP	1.43	UP	2.04	UP
TRINITY_DN980_c0_g1	uncharacterized protein LOC113820017	1.66	UP	1.58	UP	2.23	UP	1.60	UP

Note: The multiple of difference is represented by log2FC, where FC is Fold change.

Table S3. KEGG pathway enrichment of the differentially expressed genes between Control, 0.05%, 0.1%, 0.2% and 0.4%

KEGG	KEGG ID	Ratio	P value	DEGs ID
0.05%/Control				
Starch and sucrose metabolism	map00500	8/85	0.000	TRINITY_DN6325_c0_g1丨TRINITY_DN6505_c1_g1丨TRINITY_DN16841_c0_g1丨TRINITY_DN209_c1_g1丨TRINITY_DN12252_c0_g1丨TRINITY_DN1721_c0_g1丨TRINITY_DN9176_c0_g1丨TRINITY_DN1907_c0_g4
Carbohydrate digestion and absorption	map04973	4/85	0.001	TRINITY_DN209_c1_g1丨TRINITY_DN6325_c0_g1丨TRINITY_DN9176_c0_g1丨TRINITY_DN1907_c0_g4
Nitrogen metabolism	map00910	3/85	0.002	TRINITY_DN1777_c0_g1丨TRINITY_DN6383_c0_g1丨TRINITY_DN1172_c0_g1
Pancreatic secretion	map04972	6/85	0.004	TRINITY_DN6325_c0_g1丨TRINITY_DN32432_c0_g1丨TRINITY_DN1932_c0_g1丨TRINITY_DN1172_c0_g1丨TRINITY_DN5032_c0_g1丨TRINITY_DN1907_c0_g4
Galactose metabolism	map00052	3/85	0.006	TRINITY_DN1721_c0_g1丨TRINITY_DN209_c1_g1丨TRINITY_DN9176_c0_g1
RIG-I-like receptor signaling pathway	map04622	2/85	0.022	TRINITY_DN37493_c0_g1丨TRINITY_DN11882_c0_g1
Protein digestion and absorption	map04974	4/85	0.020	TRINITY_DN32432_c0_g1丨TRINITY_DN3909_c0_g1丨TRINITY_DN5032_c0_g1丨TRINITY_DN6158_c0_g1
Th1 and Th2 cell differentiation	map04658	2/85	0.026	TRINITY_DN8309_c0_g1丨TRINITY_DN1716_c0_g1
Proximal tubule bicarbonate reclamation	map04964	2/85	0.020	TRINITY_DN6383_c0_g1丨TRINITY_DN1172_c0_g1
Lysosome	map04142	5/85	0.038	TRINITY_DN1721_c0_g1丨TRINITY_DN581_c0_g1丨TRINITY_DN632_c0_g1丨TRINITY_DN3387_c0_g1丨TRINITY_DN2042_c0_g1
0.1%/Control				
Fructose and mannose metabolism	map00051	2/35	0.015	TRINITY_DN11882_c0_g1丨TRINITY_DN27888_c0_g1
Longevity regulating pathway - multiple species	map04213	2/35	0.032	TRINITY_DN18184_c0_g4丨TRINITY_DN12488_c0_g1
Starch and sucrose metabolism	map00500	2/35	0.030	TRINITY_DN16841_c0_g1丨TRINITY_DN209_c1_g1
Insulin resistance	map04931	2/35	0.026	TRINITY_DN6609_c0_g1丨TRINITY_DN10885_c0_g2
Glycosaminoglycan biosynthesis - chondroitin sulfate/dermatan sulfate	map00532	2/35	0.009	TRINITY_DN3972_c0_g1丨TRINITY_DN15725_c0_g1

Cell adhesion molecules	map04514	2/35	0.014	TRINITY_DN6609_c0_g1丨TRINITY_DN10885_c0_g2
Pancreatic secretion	map04972	3/35	0.023	TRINITY_DN1932_c0_g1丨TRINITY_DN5032_c0_g1丨TRINITY_DN7892_c0_g1
PPAR signaling pathway	map03320	3/35	0.007	TRINITY_DN2597_c0_g1丨TRINITY_DN7925_c1_g1丨TRINITY_DN14357_c0_g1
0.2%/Control				
JAK-STAT signaling pathway	map04630	7/343	0.000	TRINITY_DN6518_c0_g2丨TRINITY_DN2726_c0_g1丨TRINITY_DN9466_c0_g1 丨TRINITY_DN20840_c0_g2丨TRINITY_DN10656_c0_g1丨TRINITY_DN20318_c0_g1 丨TRINITY_DN9403_c0_g1
Prolactin signaling pathway	map04917	5/343	0.005	TRINITY_DN20318_c0_g1丨TRINITY_DN10656_c0_g1丨TRINITY_DN1256_c0_g1 丨TRINITY_DN9466_c0_g1丨TRINITY_DN9403_c0_g1
Hippo signaling pathway - multiple species	map04392	4/343	0.008	TRINITY_DN14606_c1_g1丨TRINITY_DN35828_c0_g1丨TRINITY_DN7883_c0_g1 丨TRINITY_DN9150_c0_g1
Neurotrophin signaling pathway	map04722	6/343	0.029	TRINITY_DN19143_c0_g1丨TRINITY_DN8077_c0_g1丨TRINITY_DN1256_c0_g1 丨TRINITY_DN15479_c0_g1丨TRINITY_DN10656_c0_g1丨TRINITY_DN18614_c0_g2
MicroRNAs in cancer	map05206	11/343	0.019	TRINITY_DN6518_c0_g2丨TRINITY_DN8314_c0_g1丨TRINITY_DN1246_c0_g1 丨TRINITY_DN3685_c0_g1丨TRINITY_DN3739_c0_g1丨TRINITY_DN1306_c1_g1 丨TRINITY_DN9403_c0_g1丨TRINITY_DN555_c0_g1丨TRINITY_DN15479_c0_g1 丨TRINITY_DN6609_c0_g1丨TRINITY_DN20840_c0_g2
Acute myeloid leukemia	map05221	4/343	0.021	TRINITY_DN10656_c0_g1丨TRINITY_DN6518_c0_g2丨TRINITY_DN1813_c0_g1 丨TRINITY_DN20840_c0_g2
AGE-RAGE signaling pathway in diabetic complications	map04933	6/343	0.026	TRINITY_DN3739_c0_g1丨TRINITY_DN4855_c0_g2丨TRINITY_DN20840_c0_g2 丨TRINITY_DN15479_c0_g1丨TRINITY_DN10656_c0_g1丨TRINITY_DN9030_c0_g3
ABC transporters	map02010	4/343	0.028	TRINITY_DN555_c0_g1丨TRINITY_DN10650_c0_g3丨TRINITY_DN1306_c1_g1 丨TRINITY_DN4735_c1_g1
AMPK signaling pathway	map04152	7/343	0.042	TRINITY_DN8314_c0_g1丨TRINITY_DN1256_c0_g1丨TRINITY_DN841_c0_g1 丨TRINITY_DN12252_c0_g1丨TRINITY_DN10656_c0_g1丨TRINITY_DN143_c0_g1 丨TRINITY_DN38868_c1_g2
Circadian rhythm - fly	map04711	2/343	0.043	TRINITY_DN8168_c0_g1丨TRINITY_DN162_c0_g2
0.4%/Control				

Prolactin signaling pathway	map04917	7/566	0.002	TRINITY_DN11337_c0_g1 | TRINITY_DN1256_c0_g1 | TRINITY_DN9466_c0_g1 | TRINITY_DN9403_c0_g1 | TRINITY_DN1918_c0_g2 | TRINITY_DN10656_c0_g1 | TRINITY_DN10208_c0_g1
Acute myeloid leukemia	map05221	7/566	0.001	TRINITY_DN6518_c0_g2 | TRINITY_DN6648_c0_g1 | TRINITY_DN20840_c0_g2 | TRINITY_DN1918_c0_g2 | TRINITY_DN1813_c0_g1 | TRINITY_DN10656_c0_g1 | TRINITY_DN10208_c0_g1
JAK-STAT signaling pathway	map04630	8/566	0.002	TRINITY_DN6518_c0_g2 | TRINITY_DN6648_c0_g1 | TRINITY_DN2726_c0_g1 | TRINITY_DN9466_c0_g1 | TRINITY_DN9403_c0_g1 | TRINITY_DN10656_c0_g1 | TRINITY_DN4861_c0_g1 | TRINITY_DN20840_c0_g2
Spinocerebellar ataxia	map05017	10/566	0.004	TRINITY_DN11337_c0_g1 | TRINITY_DN7604_c0_g1 | TRINITY_DN6648_c0_g1 | TRINITY_DN8077_c0_g1 | TRINITY_DN8091_c0_g1 | TRINITY_DN9320_c0_g1 | TRINITY_DN5840_c0_g1 | TRINITY_DN10656_c0_g1 | TRINITY_DN1716_c0_g3 | TRINITY_DN1716_c0_g1
MicroRNAs in cancer	map05206	17/566	0.007	TRINITY_DN16089_c0_g2 | TRINITY_DN6518_c0_g2 | TRINITY_DN8314_c0_g1 | TRINITY_DN1246_c0_g1 | TRINITY_DN3685_c0_g1 | TRINITY_DN6648_c0_g1 | TRINITY_DN1306_c1_g1 | TRINITY_DN9403_c0_g1 | TRINITY_DN1918_c0_g2 | TRINITY_DN20840_c0_g2 | TRINITY_DN1122_c0_g1 | TRINITY_DN5482_c0_g3 | TRINITY_DN10208_c0_g1 | TRINITY_DN6609_c0_g1 | TRINITY_DN5890_c0_g1 | TRINITY_DN12178_c0_g2 | TRINITY_DN5750_c1_g3
Hepatitis B	map05161	11/566	0.016	TRINITY_DN11337_c0_g1 | TRINITY_DN271_c1_g1 | TRINITY_DN9030_c0_g3 | TRINITY_DN6518_c0_g2 | TRINITY_DN19143_c0_g1 | TRINITY_DN1918_c0_g2 | TRINITY_DN7502_c0_g1 | TRINITY_DN2018_c0_g1 | TRINITY_DN10656_c0_g1 | TRINITY_DN10208_c0_g1 | TRINITY_DN5890_c0_g1
Toll-like receptor signaling pathway	map04620	6/566	0.016	TRINITY_DN11337_c0_g1 | TRINITY_DN1918_c0_g2 | TRINITY_DN7502_c0_g1 | TRINITY_DN271_c1_g1 | TRINITY_DN10656_c0_g1 | TRINITY_DN10208_c0_g1
Th1 and Th2 cell differentiation	map04658	7/566	0.001	TRINITY_DN11337_c0_g1 | TRINITY_DN3685_c0_g1 | TRINITY_DN1918_c0_g2 | TRINITY_DN5750_c1_g3 | TRINITY_DN10208_c0_g1 | TRINITY_DN1716_c0_g1 | TRINITY_DN8309_c0_g1

Autophagy - animal	map04140	14/566	0.006	TRINITY_DN8020_c0_g1丨TRINITY_DN7604_c0_g1丨TRINITY_DN11337_c0_g1丨TRINITY_DN6648_c0_g1丨TRINITY_DN28994_c0_g3丨TRINITY_DN8314_c0_g1丨TRINITY_DN2957_c0_g1丨TRINITY_DN2676_c0_g1丨TRINITY_DN8091_c0_g1丨TRINITY_DN9320_c0_g1丨TRINITY_DN10208_c0_g1丨TRINITY_DN5840_c0_g1丨TRINITY_DN10656_c0_g1丨TRINITY_DN15542_c0_g1
B-cell receptor signaling pathway	map04662	6/566	0.007	TRINITY_DN3812_c0_g1丨TRINITY_DN11405_c0_g1丨TRINITY_DN4390_c0_g2丨TRINITY_DN1918_c0_g2丨TRINITY_DN10656_c0_g1丨TRINITY_DN10208_c0_g1
RIG-I-like receptor signaling pathway	map04622	5/566	0.015	TRINITY_DN37493_c0_g1丨TRINITY_DN11337_c0_g1丨TRINITY_DN1918_c0_g2丨TRINITY_DN19143_c0_g1丨TRINITY_DN7502_c0_g1
Th17-cell differentiation	map04659	6/566	0.019	TRINITY_DN11337_c0_g1丨TRINITY_DN6648_c0_g1丨TRINITY_DN3236_c0_g1丨TRINITY_DN1918_c0_g2丨TRINITY_DN10208_c0_g1丨TRINITY_DN9030_c0_g3
TNF signaling pathway	map04668	9/566	0.020	TRINITY_DN11337_c0_g1丨TRINITY_DN271_c1_g1丨TRINITY_DN1918_c0_g2丨TRINITY_DN8077_c0_g1丨TRINITY_DN7502_c0_g1丨TRINITY_DN10656_c0_g1丨TRINITY_DN10208_c0_g1丨TRINITY_DN5890_c0_g1丨TRINITY_DN6900_c0_g1
Insulin signaling pathway	map04910	12/566	0.015	TRINITY_DN11337_c0_g1丨TRINITY_DN1737_c0_g1丨TRINITY_DN8314_c0_g1丨TRINITY_DN2776_c0_g1丨TRINITY_DN6648_c0_g1丨TRINITY_DN1187_c0_g1丨TRINITY_DN12252_c0_g1丨TRINITY_DN10656_c0_g1丨TRINITY_DN10208_c0_g1丨TRINITY_DN6609_c0_g1丨TRINITY_DN4193_c0_g1丨TRINITY_DN10885_c0_g2
Signaling pathways regulating pluripotency of stem cells	map04550	8/566	0.013	TRINITY_DN6518_c0_g2丨TRINITY_DN3591_c0_g1丨TRINITY_DN2551_c0_g1丨TRINITY_DN10656_c0_g1丨TRINITY_DN10208_c0_g1丨TRINITY_DN10085_c0_g3丨TRINITY_DN17933_c0_g1丨TRINITY_DN16089_c0_g2
Longevity regulating pathway	map04211	8/566	0.013	TRINITY_DN8020_c0_g1丨TRINITY_DN8314_c0_g1丨TRINITY_DN1256_c0_g1丨TRINITY_DN1918_c0_g2丨TRINITY_DN9320_c0_g1丨TRINITY_DN5840_c0_g1丨TRINITY_DN10656_c0_g1丨TRINITY_DN6648_c0_g1
Hippo signaling pathway - multiple species	map04392	5/566	0.009	TRINITY_DN14606_c1_g1丨TRINITY_DN35828_c0_g1丨TRINITY_DN7883_c0_g1丨TRINITY_DN12964_c0_g1丨TRINITY_DN4152_c0_g1
Starch and sucrose metabolism	map00500	10/566	0.010	TRINITY_DN6325_c0_g1丨TRINITY_DN29293_c0_g1丨TRINITY_DN16841_c0_g1丨TRINITY_DN209_c1_g1丨TRINITY_DN12252_c0_g1丨TRINITY_DN1721_c0_g1丨TRINITY_DN9176_c0_g1丨TRINITY_DN4187_c0_g1丨TRINITY_DN8367_c0_g2丨TRINITY_DN1737_c0_g1

Endocrine resistance	map01522	8/566	0.012	TRINITY_DN11337_c0_g1丨TRINITY_DN5064_c0_g1丨TRINITY_DN6648_c0_g1丨TRINITY_DN3685_c0_g1丨TRINITY_DN5750_c1_g3丨TRINITY_DN10656_c0_g1丨TRINITY_DN10208_c0_g1丨TRINITY_DN8309_c0_g1
Osteoclast differentiation	map04380	6/566	0.013	TRINITY_DN11337_c0_g1丨TRINITY_DN11405_c0_g1丨TRINITY_DN1918_c0_g2丨TRINITY_DN10656_c0_g1丨TRINITY_DN10208_c0_g1丨TRINITY_DN9030_c0_g3
Glycosaminoglycan biosynthesis - chondroitin sulfate/dermatan sulfate	map00532	6/566	0.026	TRINITY_DN5242_c0_g1丨TRINITY_DN24399_c0_g1丨TRINITY_DN10467_c0_g1丨TRINITY_DN3972_c0_g1丨TRINITY_DN371_c0_g1丨TRINITY_DN1291_c0_g1
T-cell receptor signaling pathway	map04660	6/566	0.036	TRINITY_DN11337_c0_g1丨TRINITY_DN3812_c0_g1丨TRINITY_DN4390_c0_g2丨TRINITY_DN1918_c0_g2丨TRINITY_DN10656_c0_g1丨TRINITY_DN10208_c0_g1
Sphingolipid signaling pathway	map04071	10/566	0.042	TRINITY_DN11337_c0_g1丨TRINITY_DN1918_c0_g2丨TRINITY_DN8077_c0_g1丨TRINITY_DN11513_c0_g1丨TRINITY_DN27382_c0_g1丨TRINITY_DN521_c0_g2丨TRINITY_DN10656_c0_g1丨TRINITY_DN10208_c0_g1丨TRINITY_DN27926_c0_g1丨TRINITY_DN5802_c0_g1
Cholesterol metabolism	map04979	7/566	0.031	TRINITY_DN34912_c0_g7丨TRINITY_DN30140_c0_g1丨TRINITY_DN2042_c0_g1丨TRINITY_DN6495_c1_g1丨TRINITY_DN5555_c0_g1丨TRINITY_DN41_c0_g1丨TRINITY_DN1173_c0_g1
Chagas disease	map05142	7/566	0.031	TRINITY_DN11337_c0_g1丨TRINITY_DN271_c1_g1丨TRINITY_DN7502_c0_g1丨TRINITY_DN1918_c0_g2丨TRINITY_DN10656_c0_g1丨TRINITY_DN10208_c0_g1丨TRINITY_DN9030_c0_g3
Neurotrophin signaling pathway	map04722	8/566	0.037	TRINITY_DN11337_c0_g1丨TRINITY_DN19143_c0_g1丨TRINITY_DN8077_c0_g1丨TRINITY_DN1256_c0_g1丨TRINITY_DN1187_c0_g1丨TRINITY_DN1918_c0_g2丨TRINITY_DN10656_c0_g1丨TRINITY_DN10208_c0_g1
Type II diabetes mellitus	map04930	4/566	0.028	TRINITY_DN11337_c0_g1丨TRINITY_DN10208_c0_g1丨TRINITY_DN1737_c0_g1丨TRINITY_DN6648_c0_g1
Kaposi sarcoma-associated herpesvirus infection	map05167	10/566	0.035	TRINITY_DN11337_c0_g1丨TRINITY_DN271_c1_g1丨TRINITY_DN6518_c0_g2丨TRINITY_DN6648_c0_g1丨TRINITY_DN7502_c0_g1丨TRINITY_DN8091_c0_g1丨TRINITY_DN1918_c0_g2丨TRINITY_DN10656_c0_g1丨TRINITY_DN10208_c0_g1丨TRINITY_DN5890_c0_g1
Fc epsilon RI signaling pathway	map04664	5/566	0.034	TRINITY_DN10656_c0_g1丨TRINITY_DN11337_c0_g1丨TRINITY_DN4390_c0_g2丨TRINITY_DN10208_c0_g1丨TRINITY_DN271_c1_g1

Carbohydrate digestion and absorption	map04973	6/566	0.041	TRINITY_DN6325_c0_g1丨TRINITY_DN1737_c0_g1丨TRINITY_DN209_c1_g1丨TRINITY_DN10656_c0_g1丨TRINITY_DN9176_c0_g1丨TRINITY_DN8367_c0_g2
Dorso-ventral axis formation	map04320	6/566	0.041	TRINITY_DN16218_c0_g1丨TRINITY_DN3685_c0_g1丨TRINITY_DN7144_c0_g1丨TRINITY_DN5750_c1_g3丨TRINITY_DN10208_c0_g1丨TRINITY_DN91_c0_g1
Breast cancer	map05224	8/566	0.046	TRINITY_DN6518_c0_g2丨TRINITY_DN6648_c0_g1丨TRINITY_DN3685_c0_g1丨TRINITY_DN5750_c1_g3丨TRINITY_DN2551_c0_g1丨TRINITY_DN10656_c0_g1丨TRINITY_DN10208_c0_g1丨TRINITY_DN8309_c0_g1
Glycosaminoglycan biosynthesis - keratan sulfate	map00533	3/566	0.031	TRINITY_DN371_c0_g1丨TRINITY_DN24399_c0_g1丨TRINITY_DN10467_c0_g1
ABC transporters	map02010	5/566	0.040	TRINITY_DN5294_c0_g1丨TRINITY_DN10764_c0_g1丨TRINITY_DN10650_c0_g3丨TRINITY_DN1306_c1_g1丨TRINITY_DN4735_c1_g1
Cushing syndrome	map04934	10/566	0.046	TRINITY_DN1311_c0_g1丨TRINITY_DN16940_c0_g9丨TRINITY_DN23062_c0_g6丨TRINITY_DN6472_c0_g1丨TRINITY_DN23062_c0_g3丨TRINITY_DN6495_c1_g1丨TRINITY_DN10208_c0_g1丨TRINITY_DN1637_c0_g1丨TRINITY_DN2551_c0_g1丨TRINITY_DN8192_c0_g1
